# Canine Macrophage DH82 Cell Line As a Model to Study Susceptibility to *Trypanosoma cruzi* Infection

**DOI:** 10.3389/fimmu.2017.00604

**Published:** 2017-05-31

**Authors:** Pedro Henrique Braz Mendonça, Raphael Francisco Dutra Barbosa da Rocha, Julliane Brito de Braz Moraes, Isabel Ferreira LaRocque-de-Freitas, Jorgete Logullo, Alexandre Morrot, Marise Pinheiro Nunes, Celio Geraldo Freire-de-Lima, Debora Decote-Ricardo

**Affiliations:** ^1^Instituto de Veterinária, Universidade Federal Rural do Rio de Janeiro, Rio de Janeiro, Brazil; ^2^Instituto de Biofísica Carlos Chagas Filho, Universidade Federal do Rio de Janeiro, Rio de Janeiro, Brazil; ^3^Instituto de Microbiologia Paulo de Góes, Universidade Federal do Rio de Janeiro, Rio de Janeiro, Brazil; ^4^Instituto Oswaldo Cruz, FIOCRUZ, Rio de Janeiro, Brazil

**Keywords:** *Trypanosoma cruzi*, chagas disease, dogs, macrophages, immunomodulation, infection model, susceptibility

## Abstract

*Trypanosoma cruzi* is an obligatory intracellular protozoan parasite, and it is the etiological agent of Chagas’ disease that is endemic in the Americas. In addition to humans, a wide spectrum of mammals can be infected by *T. cruzi*, including dogs. Dogs develop acute and chronic disease, similar to human infection. *T. cruzi* can infect almost all cell types and after cell invasion, the metacyclics trypomastigotes localize in the cytoplasm, where they transform into amastigotes, the replicative form of *T. cruzi* in mammals. After amastigote multiplication and differentiation, parasites lyse host cells and spread through the body by blood circulation. In this work, we evaluated the *in vitro* ability of *T. cruzi* to infect a canine macrophage cell line DH82 compared with RAW264.7, a murine tissue culture macrophage. Our results have shown that the *T. cruzi* is able to infect, replicate and differentiate in DH82 cell line. We observed that following treatment with LPS and IFN-γ DH82 cells were more resistant to infection and that resistance was not related reactive oxygen species production in our system. In this study, we also found that DH82 cells became more susceptible to *T. cruzi* infection when cocultured with apoptotic cells. The analysis of cytokine production has showed elevated levels of the TGF-β, IL-10, and TNF-α produced by *T. cruzi*-infected canine macrophages. Additionally, we demonstrated a reduced expression of the MHC class II and CD80 by infected DH82 cell line.

## Introduction

Chagas’ disease is caused by the protozoan hemoflagellate *Trypanosoma cruzi*, which is transmitted by a blood-sucking reduviid bugs (1). Although it was described more than 100 years ago, this disease remains a serious public health problem in South and Central America countries, which come to account for 12 million to 14 million of infected people (2).

The *T. cruzi* infection begins when the infective metacyclic trypomastigotes forms are transmitted to humans or other mammals by the infected feces of triatomine bugs. Trypomastigotes are able to invade cells, phagocytic or non-phagocytic, differentiate, and multiply in almost all mammalian nucleated cells, including macrophages, muscle cells, glial cells, neurons, fibroblasts, adipocytes, and endothelial cells. After cell invasion trypomastigotes transform into amastigotes, that multiply intensively, then transform into trypomastigotes and finally break the host-infected cells. After reaching the bloodstream and lymphatic vessels, blood trypomastigotes can invade other tissues, penetrate the cells, and differentiate again into amastigotes, forming new multiplying foci. Due to the cyclical sequence of these events, a rapid increase of circulating trypomastigotes is observed. Thus, during the acute phase of Chagas’ disease parasitemia is readily detectable by fresh blood examination. After the acute phase, there is a latent phase of infection, also called indeterminate phase, which can last for long periods of time or can be permanent, then develops into a chronic stage (3).

*Trypanosoma cruzi* can infect a wide range of wild and domestic mammals that serve as parasite reservoirs. Dogs play an important role in domestic cycle of infection representing a risk factor for humans (4, 5). Naturally infected dogs or seropositive dogs were detected in the United States (6), Mexico, Argentina, Venezuela, and Panama (7, 8). In Brazil, the presence of infected animals was also reported in different areas of the country (5, 9). The canine Chagas’ disease is becoming a major veterinarian concern in the Americas (8).

*Trypanosoma cruzi*-infected dogs reproduce several aspects of the infection observed in humans. In the acute phase of infection, dogs develop lymphadenopathy and hepatosplenomegaly and show circulating blood trypomastigotes (10). In animals that progress to the chronic phase of infection, cardiac involvement with cardiomegaly occurs (11). Recently, it was reported that seropositive dogs exhibit high levels of IgG that correlates with the severity of myocarditis, characterized by mononuclear cell infiltrates (12). In experimentally infected dogs of Beagle breed, it was observed high levels of proinflammatory cytokines such as INF-γ, TNF-α, and low IL-10 levels in the acute phase of infection. These data suggest that the development of chronic heart abnormalities may be related to a strong Th1-type response during the acute phase (10). Using Beagle dogs infected by two distinct genetic groups of parasites, Guedes and coworkers (10) showed that the cardiac type 1/2 chemokines and their receptors expression depend on the genetic diversity of parasites that can determine the migration of Th1 or Th2 cells to the heart during the acute and chronic phases of the disease. In addition, they have showed that infected dogs that develop the cardiac form of disease, have increased expression of CCL5, CCL4, and CXCR3 type 1 chemokines receptors when compared with dogs in the indeterminate phase of the disease (13).

*Trypanosoma cruzi* invasion strategy demonstrates its ability to subvert the macrophage antimicrobial defense mechanisms. The trypomastigotes interact with host cells by inducing a signal that results in lysosomes recruitment in a Ca^+^-dependent manner. Initially, parasites enter the cells and are located inside parasitophorous vacuoles, at that time *T. cruzi* releases a hemolysin called TcTox to able escape into the host cell cytoplasm (14).

The intracellular signaling of macrophages can be triggered by the innate immunity receptor is also explored by *T. cruzi*. Parasite-derived molecules, like trypomastigote-derived glycosylphosphatidylinositol anchors of mucin-like glycoproteins, can be recognized by toll-like receptors (TLR2) and initiate a cascade of signals that induces the production of IL-12, TNF-α, and nitric oxide (NO) (15–17). The *T. cruzi* GPI-mucin can induce a state of tolerance phenomena in macrophages *in vitro*, similar to that observed when macrophages were stimulated by LPS (18). These researchers showed that macrophages stimulated with low doses of LPS have a lock response to a second stimulus with LPS. This resulted in suppression of IL-12 and TNF-α production, while the production of NO and IL-10 was not affected.

These mechanisms of tolerance induced by endotoxin interfere with the signaling pathways mediated by TLR4, resulting in decreased activation of MAPK and the p50 subunit NFκB. It has been shown that stimulation of macrophages by LPS then mucin-GPI *T. cruzi* produces a tolerogenic effect associated with defects in phosphorylation of IRAK-1, MAPK activation, and degradation IκB (19). An important aspect of these data is the fact that TLR activation can lead to suppression of another TLR, resulting in blocking the activation of macrophages (20). Thus, the interaction between the parasite and the host cell could modulate the activation process that favors the invasion and persistence of the parasite.

Besides, to directly affect macrophages, infection with *T. cruzi* can produce a modulating effect through the deleterious events affecting other cell populations. An important feature observed during experimental infection with *T. cruzi* is an intense CD4^+^ T cell death by apoptosis (21). The interaction of macrophages with apoptotic bodies leads to TGF-β production that induces the deactivation of macrophages making it more susceptible to infection (22).

Various studies have used cell culture systems derived from mammals to understand the interaction between the host and different *T. cruzi* strains. Spleen and peritoneal macrophages obtained from a variety of mouse strains have been widely used to study *T. cruzi* infectivity *in vitro*. Although the importance of dogs as domestic reservoir, as a model for testing of vaccines and new drugs for the treatment of Chagas’ disease is recognized, there is a lack of experimental studies with dog macrophages. Here we have investigated the *in vitro* infectivity rate of trypomastigotes and the replication of amastigotes of *T. cruzi* Dm28c in dog DH82 and mouse RAW264.7 tissue culture macrophages. The DH82 cell is a line isolated from a dog presenting malignant histiocytosis, this lineage shows macrophages morphology and is excellent phagocytic cells. Also, express in the cell surface Fc-gamma receptor and are negative for the C3b receptor (23).

In the present study, we demonstrated that DH82 cells are susceptible to *T. cruzi* infection and the parasites were able to modulate expression of the MHC class II, CD80, and cytokines production.

## Materials and Methods

### Cell Culture

To prepare tissue culture cells for infection, DH82 and RAW264.7 cells (ATCC) were grown in 75 cm^2^ tissue culture flasks (T75) (Nunc, Roskilde, Denmark) with Dulbecco’s Modified Eagle Medium (DMEM; Sigma-Aldrich) supplemented with 10% fetal calf serum (FCS; Gibco), 100 μg/mL of streptomycin and 100 Units/mL of penicillin, 1% MEM non-essential amino acids (Sigma-Aldrich), and 2 mM of l-glutamine were cultured at 37°C with 5% CO_2_. Subcultures of both cell lines were carried out once a week, when they reached a confluence of 95–100%. Cells were released with 0.25% of trypsin (Difco) and 1 mM of EDTA (Sigma-Aldrich), harvested, and washed twice in HBSS by centrifugation at room temperature for 10 min at 250*g*. The supernatant was discarded, cell pellet was resuspended in DMEM containing 10% FCS, cultured into new T75 flasks, and culture medium was changed after 3 days in culture. Prior to use in infectivity assays, cells’ viability was assessed by trypan blue exclusion in a hemocytometer.

### Parasites and Infection

Infection of DH82 and RAW264.7 cell lines were carried out in 24-well plates at a concentration of 2.0 × 10^5^ macrophages/well. Cells were infected overnight with chemically induced metacyclic trypomastigotes forms of *T. cruzi* clone Dm28c, obtained as previously described (24), at a 1:1, 3:1, and 5:1 parasite-to-cell ratios in 1 mL of DMEM 10% FCS and incubated at 37°C in 5% CO_2_. In the following day (day 1), monolayers were extensively washed to remove extracellular parasites and cultured with complete culture medium containing 1% Nutridoma (Sigma-Aldrich) instead of FCS. Some cultures were stimulated with 400 ng/mL of lipopolysaccharides from *Salmonella enterica* serotype typhimurium (LPS, Sigma-Aldrich) and 1.5 ng/mL of recombinant canine or murine IFN-γ (Serotech) 24 h after infection. DH82 and RAW264.7 were also seeded in 24-well plate containing glass coverslips for parasite burden evaluation. Three days after infection, coverslips were washed with HBSS, fixed with methanol, and stained with Diff-Quick (Thermo Fisher, Waltham, MA, USA). Amastigotes were counted at 100× oil immersion on a (Olympus) microscope. The number of amastigotes was estimated in 100 infected cells per coverslip, and the frequency of infection was compared among six coverslips per time point. The number of viable trypomastigotes released in the supernatants was evaluated each 2 days of culture for 9 days, using a Neubauer chamber. We also conducted experiments using *T. cruzi* of CL strain transfected with green fluorescent protein (GFP) (25) obtained from Vero cells. The DH82 cells were infected overnight (ON). After that, the monolayers were washed, and DH82 cells were stained with DAPI 1.5 μg/mL (VECTASHIELD^®^) at room temperature for 30 min before examination by confocal microscopy Zeiss Axioplan.

### Detection of Reactive Oxygen Species (ROS)

Intracellular levels of ROS were quantified by oxydation of non-fluorescent 2′,7′-dichlorofluorescein probe, delivered as diacetate form (DCFH-DA), to the fluorescent product 2′,7′-dichlorofluorescein (26). DH82 cells were seeded in 96-well plate (5.0 × 10^4^ cells/well), infected with metacyclic trypomastigotes at a ratio of 5:1 (parasite/cell), stimulated or not with LPS (400 ng/mL, Sigma-Aldrich) and recombinant IFN-γ (1.5 ng/mL, Serotech) and after 1, 2, 3, and 4 h later cells were washed and loaded with 10 μM DCFH-DA (Invitrogen) for 20 min at 37°C. Uninfected cells were used as negative control. In different checkpoints, the cells were washed, and fluorescence was measured (excitation = 485 nm excitation; λ emission = 535 nm) in an FLx800 Fluorescence Microplate Reader (BioTek).

### Detection of Nitric Oxide (NO)

Quantification of NO DH82 and RAW264.7 (2.5 × 10^5^/mL) was cultivated in the presence of LPS (200 or 400 ng/mL) and INF-γ (1.5 ng/mL). After 12 and 24 h of incubation, NO production was evaluated by the presence of the nitrite accumulated in the supernatant of cultures using Griess colorimetric method.

### Apoptotic Lymphocytes and Coculture with DH82

In order to evaluate the effect of apoptotic lymphocytes in *T. cruzi* growth *in vitro*, we have used two approaches. In both approaches, DH82 macrophages were seeded into 24-well plate at a concentration of 2.0 × 10^5^ cells/well, incubated for 1 h at 37°C in a humidified atmosphere with 5% CO_2_ and the adherent monolayer was washed to remove non-adherent cells. Apoptotic cells were prepared as previously described (27). Briefly, single cell suspension of the Jurkat human lymphoblastoid cells was prepared and irradiated with 30 Gy (Apo-2) as previous described (27). We first performed experiments with *T. cruzi*-infected DH82 monolayers at a 5:1 parasite/cell ratio. Extracellular parasites were removed by washing the wells with HBSS and then apoptotic lymphocytes were added to the cultures at a 5:1 apoptotic lymphocyte:macrophage ratio, and kept at 37°C with 5% CO_2_ for 9 days. In other experiments, prior to *T. cruzi* infection, the DH82 monolayer were incubated with apoptotic lymphocytes at a 5:1 apoptotic lymphocyte:macrophage ratio for 2 h at 37°C with 5% CO_2_. After that period non-internalized apoptotic cells were removed and adherent macrophages were infected with *T. cruzi* at 5:1 parasite:macrophage ratio for ON at 37°C. After removing free parasites, the medium was replaced and infected DH82 cells were further incubated at 37°C with 5% CO_2_. In both experiments, the extracellular motile trypomastigotes were determined in cell supernatants after 9 days in culture by counting parasites in Neubauer chambers (28).

### Cytokine Determination

Cell supernatants were collected at 24 h postinfection for cytokine determination. TNF-α, TGF-β, and IL-10 concentration was estimated by the method of sandwich immunoassay (ELISA) according to methodology recommended by the manufacturer (R&D). The optical density was evaluated by reading in a microplate spectrophotometer (Versamax Microplates Reader Molecular Devices, USA), with filter of 405 nm.

### Flow Cytometric Analysis

To assess the expression of MHC class II and CD80 on DH82 macrophage cell line by flow cytometric analysis, cells were infected or not with *T. cruzi* Dm28c and were activated or not in the presence of recombinant IFN-γ and LPS. Cells were detached at 24 h postinfection, washed, adjusted to a concentration of 5.0 × 10^5^ cells/tube and blocked with anti-CD16/CD32 (Calbiochem) at concentration of the 1 μg/10^6^ cells to prevent non-specific antibody binding to Fc-receptors. Cells were stained with MHC class II-FITC, and CD80-PE antibodies (Serotech). All washing steps were performed with phosphate-buffered saline containing 3% bovine serum albumin and 0.02% of sodium azide. Data were acquired (10,000 events), evaluated on FACSCalibur™ cytometer, and analyzed using Cell-Quest^®^ software (BD Biosciences, Heidelberg, Germany). Analyzed gates of scattered dot plots were adjusted not to exceed 2% of positive staining cells related to the particular negative controls (29).

### Statistical Analysis

Statistical analysis was performed in the program GraphPad InStat version 3.01 (San Diego, USA). Data were analyzed by the method of Student’s *t*-test. Differences at *p*-value 0.05 or lower were reported as significant for a given comparison.

## Results

### DH82 Canine Macrophages Are Infected by *T. cruzi*

**Figure 1:**
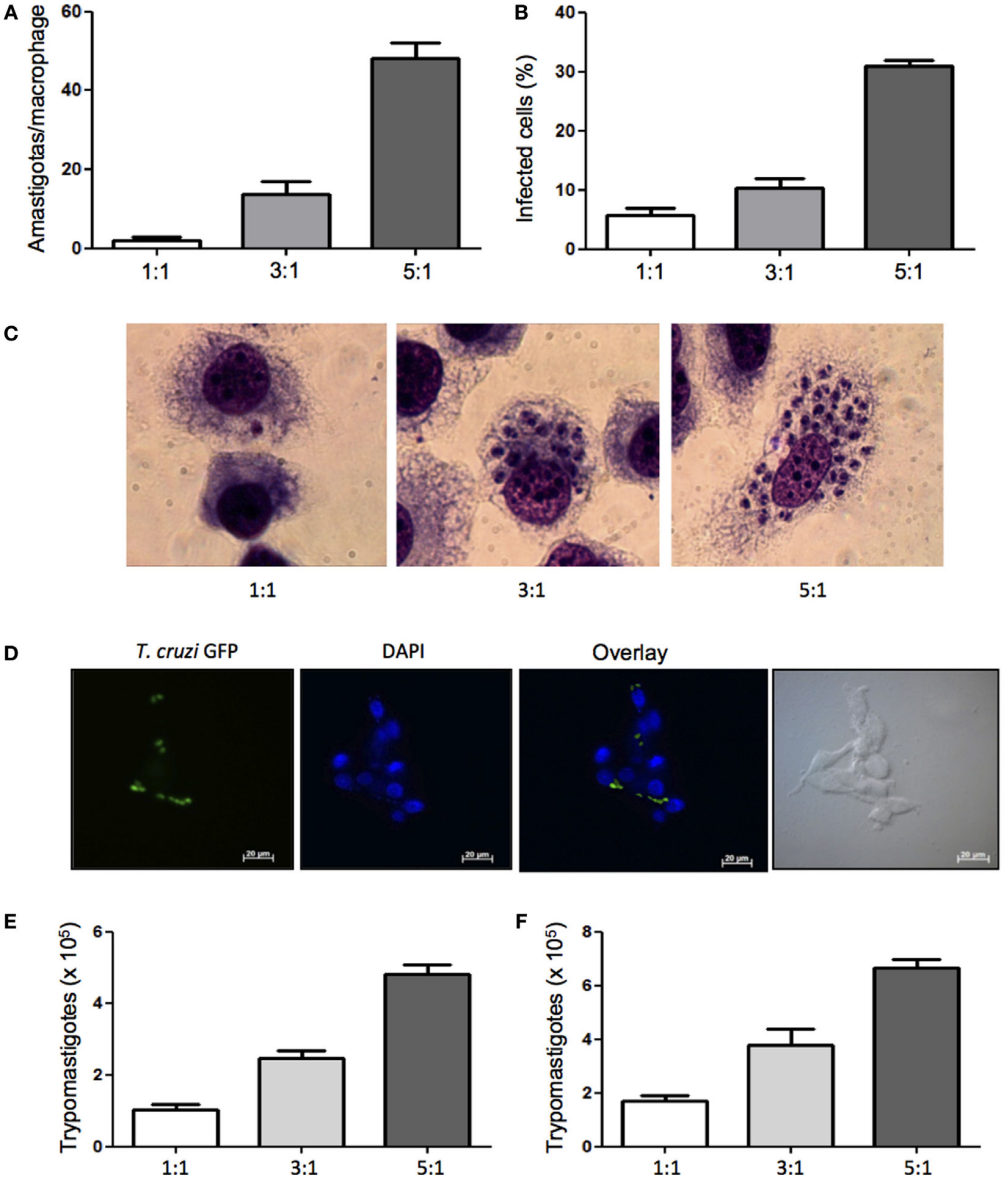
**Infectivity of *Trypanosoma cruzi* in canine macrophage DH82 cell line**. DH82 cells were cultured (2.5 × 10^5^/mL) with trypomastigotes forms of *T. cruzi* Dm28c clone. After overnight incubation, the cell culture was washed, and phagocyte was cultured for another 3 days with Dulbecco’s Modified Eagle Medium (DMEM) at 37°C. After this period, cells were stained and amastigotes inside the macrophages were counted under the light microscope **(A)** and set the percentage of infected cells **(B)**. Infected macrophage displaying amastigotes after 3 days of infection with *T. cruzi* staining with Diff-Quick **(C)** and infection with CL strain green fluorescent protein (GFP) **(D)** observed by confocal microscope. To quantify trypomastigotes forms in the supernatants, the cells were infected with trypomastigotes forms of *T. cruzi* Dm28c clone. After overnight incubation the cell culture was washed and phagocyte were cultured for another 9 days with DMEM at 37°C. The trypomastigotes forms were quantified in the supernatant of the cultures of infected DH82 macrophages after 7 days **(E)** and 9 days **(F)**. All cultures were performed in triplicate and bars show the mean + SD.

Previously published studies report *T. cruzi* infection of various mouse macrophage cell lines to study the infectivity of *T. cruzi* strains *in vitro* (30), but infection of dog macrophages has not been showed. In order to evaluate the susceptibility of DH82, a canine macrophage cell line, to *T. cruzi* Dm 28c infection, cells were plated and infected at different parasite:cell ratios. We found that canine macrophages were infected and after 5 days in culture parasite intracellular multiplication was observed (Figures 1A–C). Analyzing the rate of infected cells, we found that 33% of DH82 cells were infected at a parasite-to-cell ratio of 5:1. (Figure 1D). After 7 days in culture a large number of trypomastigotes were counted in the supernatants (Figures 1E,F). To further confirm DH82 macrophage cell line infection, we have also used *T. cruzi* of CL strain transfected with GFP and observed the adhesion, internalization (data not shown) and differentiation into intracellular amastigotes 5 days after infection (Figure 1C). Our results showed, for the first time, that the DH82, a canine macrophage cell line is susceptible to infection with *T. cruzi*, being able to provide an enabling environment for replication and differentiation of the parasite and the release of viable infective forms.

### Canine Macrophages DH82 Are More Susceptible to Infection by *T. cruzi* than Murine Macrophage RAW264.7

**Figure 2:**
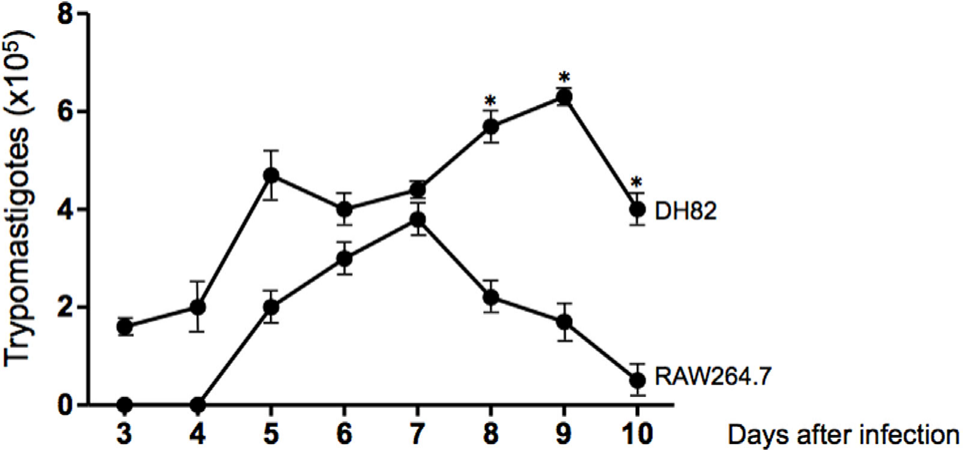
**Curve of growth of *Trypanosoma cruzi* in DH82 and RAW264.7 cell line**. DH82 and RAW 264.7 macrophages were cultured (2.5 × 10^5^/mL) with trypomastigotes forms of *T. cruzi* Dm28c clone. After overnight incubation, the cell culture was washed, and phagocyte was cultured for another 3 days with Dulbecco’s Modified Eagle Medium (DMEM) at 37°C. After 3 days, we start the quantification of the trypomastigotes forms in the supernatants of the cultures. All cultures were performed in triplicate and bars show the mean + SD. Statistical analysis was performed by *t*-test from representative results of three similar experiments (*p ≤ 0.05).

Since we have found that dog DH82 tissue culture macrophages could be infected with *T. cruzi* Dm28c, we decided to compare the rate of infection, the replication of amastigotes, and the number of trypomastigotes released in cultures of dog DH82 and mouse RAW264.7 macrophages. For this purpose, DH82 cells and RAW264.7 cells were infected at a parasite/cell ratio of 10:1 and cultivated for 9 days. At the first 3 days of infection, motile trypomastigotes forms were observed in supernatants of DH82 macrophages cell line, but not in RAW264.7 cells. After 8 to 10 days postinfection, it was observed a higher number of trypomastigotes in DH82 culture supernatants than in RAW264.7. It is important to point out that canine DH82 cells infected with *T. cruzi* were able to support the infection for the entire infection period. In contrast, *T. cruzi* replication inside murine macrophage RAW264.7 cells died 7 days after infection whereas the DH82 cells showed loss of viable morphology after 9 days of infection. These findings indicate that DH82 cells are more susceptible to infection by *T. cruzi* than RAW264.7 cell line (Figure 2).

### Activation of the DH82 Macrophage Decrease the Infectivity of *T. cruzi*

**Figure 3:**
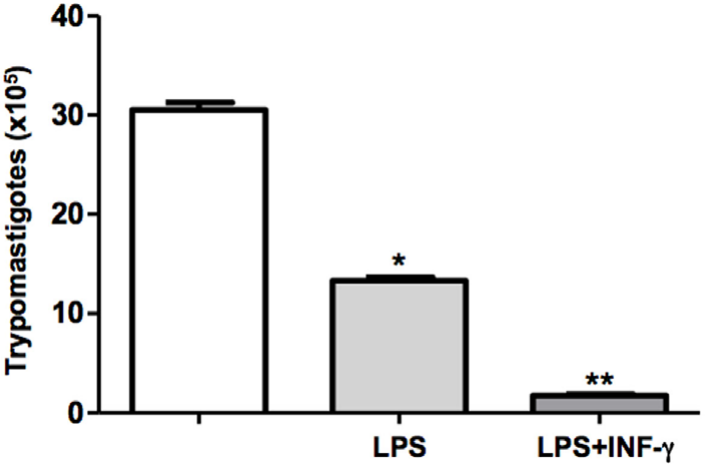
**Activated DH82 macrophages decreased trypomastigotes release**. DH82 macrophages were cultured (2.5 × 10^5^/mL). The macrophages were infected with *Trypanosoma cruzi* Dm28c clone. Some cultures were stimulated with LPS (400 ng/mL) and INF-γ (1.5 ng/mL) for 24 h. After 9 days of infection was quantified the number of the trypomastigotes forms in the supernatants of the cultures. All cultures were performed in triplicate and bars show the mean + SD. Statistical analysis was performed by *t*-test from representative results of three similar experiments (*p ≤ 0.05).

The outcome of infection is determined by the activation status of macrophages. In the present study, we have used classical macrophage activators such as LPS and INF-γ. Therefore, DH82 cells were plated, pretreated or not with LPS or LPS + IFN-γ, and were infected with *T. cruzi*. Following incubation for 9 days, the trypomastigotes forms released in the supernatants were counted. We demonstrated that LPS stimulated macrophages showed a reduced parasite burden in 50%. Furthermore, we demonstrated that pretreatment of mouse macrophages with LPS and IFN-γ resulted in low levels of macrophage infection by *T. cruzi*, suggesting that previous macrophage stimulation was able to effectively control parasite replication (Figure 3).

### Reactive Species of Oxygen Produced by DH82 Macrophages Is Not Responsible for the Decrease to Infection

**Figure 4:**
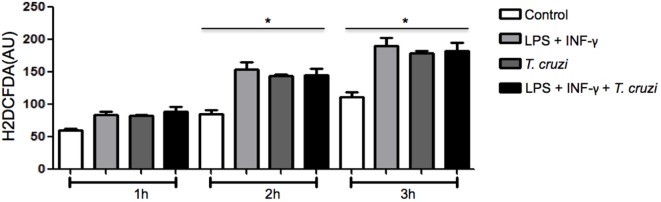
**The production of reactive species of oxygen (ROS) does not affect the infectivity of *Trypanosoma cruzi* in DH82 cells**. DH82 macrophages were cultured (5.0 × 10^4^/mL) and incubated with H2DCFDA, followed by washing and some cultures were stimulated with LPS (400 ng/mL) or LPS and INF-γ (1.5 ng/mL) in indicated time. All cultures were performed in triplicate are shown as the mean arbitrary fluorescence units (AU) + SD. Statistical analysis was performed by *t*-test from representative results of three similar experiments (*p ≤ 0.05).

Since ROS production is a crucial event associated with *in vitro* microbicidal activity in macrophages (17), we measured the production of ROS to evaluate if it could interfere in intracellular parasite replication of DH82 cells infected by *T. cruzi*. The amount of ROS produced was measured in cultures that were infected or not, in the presence or absence of LPS and IFN-γ. Increased ROS production was similar in activated and non-activated infected cells as well as in uninfected and activated cells (Figure 4). Taking into consideration that stimulation of DH82 canine macrophages with LPS and IFN-γ restricted intracellular replication and ROS production was elevated, associated with the fact that ROS release was also observed in *T. cruzi*-infected and unactivated DH82 cells at similar level suggests that, in our system, ROS is not involved in limiting parasite replication inside DH82 cells infected by *T. cruzi*.

### Profile of Cytokines of DH82 Cells after Infection with *T. cruzi*

**Figure 5:**
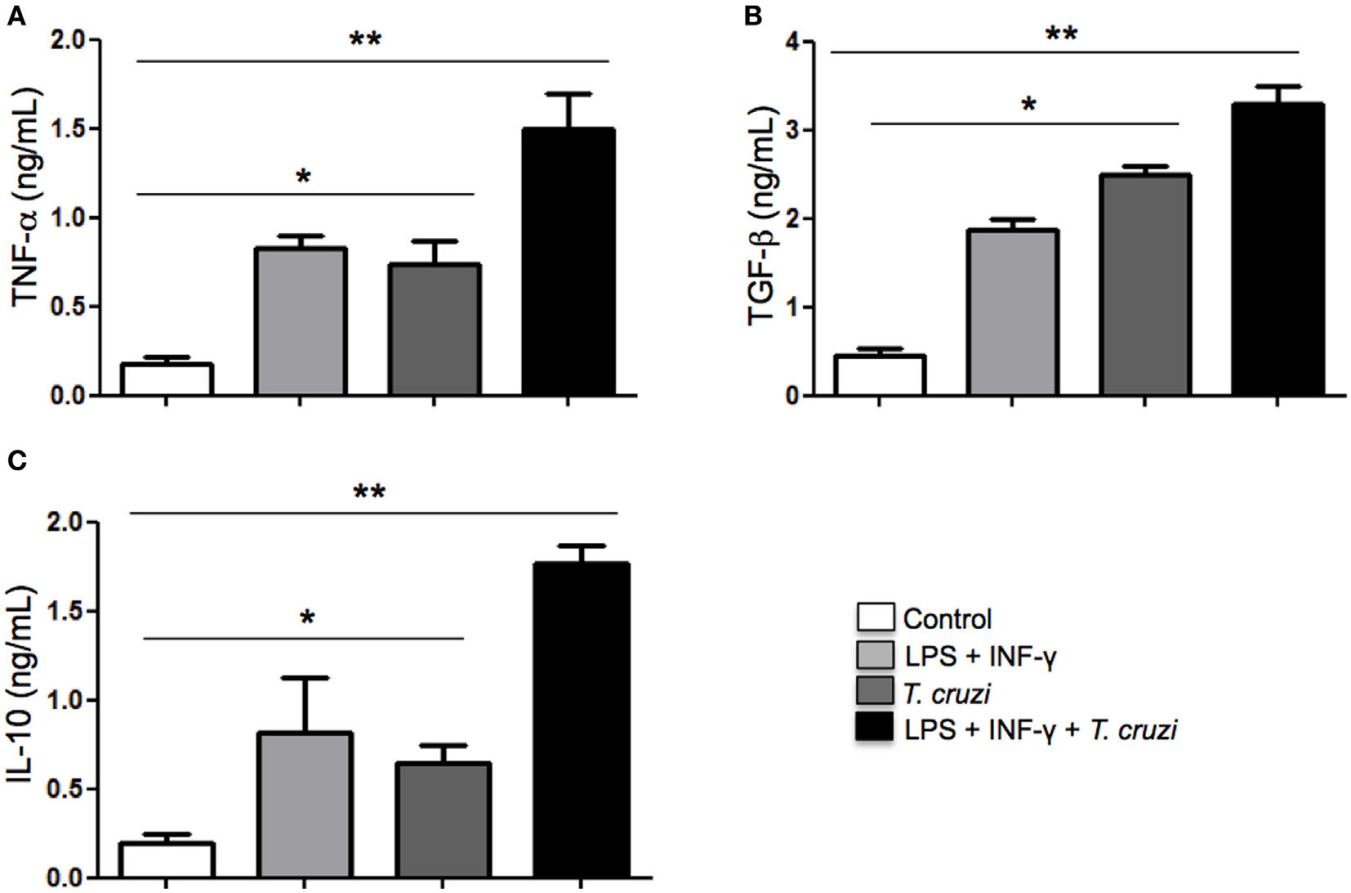
**Modulation of cytokines production in DH82 cells by *Trypanosoma cruzi* infection**. DH82 macrophages (2.5 × 10^5^/mL) were infected with trypomastigotes forms of *T. cruzi* Dm28c clone. After infection the cells were washed and some cultures were stimulated with LPS (400 ng/mL) and INF-γ (1.5 ng/mL). After 24 h, the supernatant was collected and TNF-α **(A)**, TGF-β **(B)**, and IL-10 **(C)** were measured by ELISA. All cultures were performed in triplicate and bars show the mean + SD. Statistical analysis was performed by *t*-test from representative results of three similar experiments (*p ≤ 0.05, **p ≤ 0.01).

Cytokines play a fundamental role in mediating interaction between macrophages, T lymphocytes, and other cells from the immune system in order to establish an immune response to control disease progression or in maintaining a persistent infection. Depending on the type of cytokines produced, intracellular parasites can be eliminated or can favor its permanence in the infected host (31). Here, we analyzed the TNF-α, TGF-β, and IL-10 cytokines production in the supernatants of *T. cruzi*-infected DH82 cells after overnight stimulation or not with LPS and IFN-γ. Our results showed that secretion of TNF-α production was detected in *T. cruzi*-infected DH82 macrophages at similar levels observed in stimulated macrophages (Figure 5A). However, with stimulation by LPS and IFN-γ, infected cells exhibited TNF-α production and reached higher levels as seen in Figure 5A. With regard to IL-10, maximal production was also detected in stimulated and infected DH82 macrophages (Figure 5C). LPS and IFN-γ treatment of DH82 resulted in TGF-β secretion but *T. cruzi*-infected and stimulated macrophages triggered a substantially higher level of release TGF-β in DH82 cell than was observed with in *T. cruzi*-infected macrophages only (Figure 5B). The increased TGF-β production observed in infected DH82 cells indicate that TGF-β might be involved in the success of DH82 cell infection by *T. cruzi*.

### MHC Class II and CD80 Are Modulated after Infection

**Figure 6:**
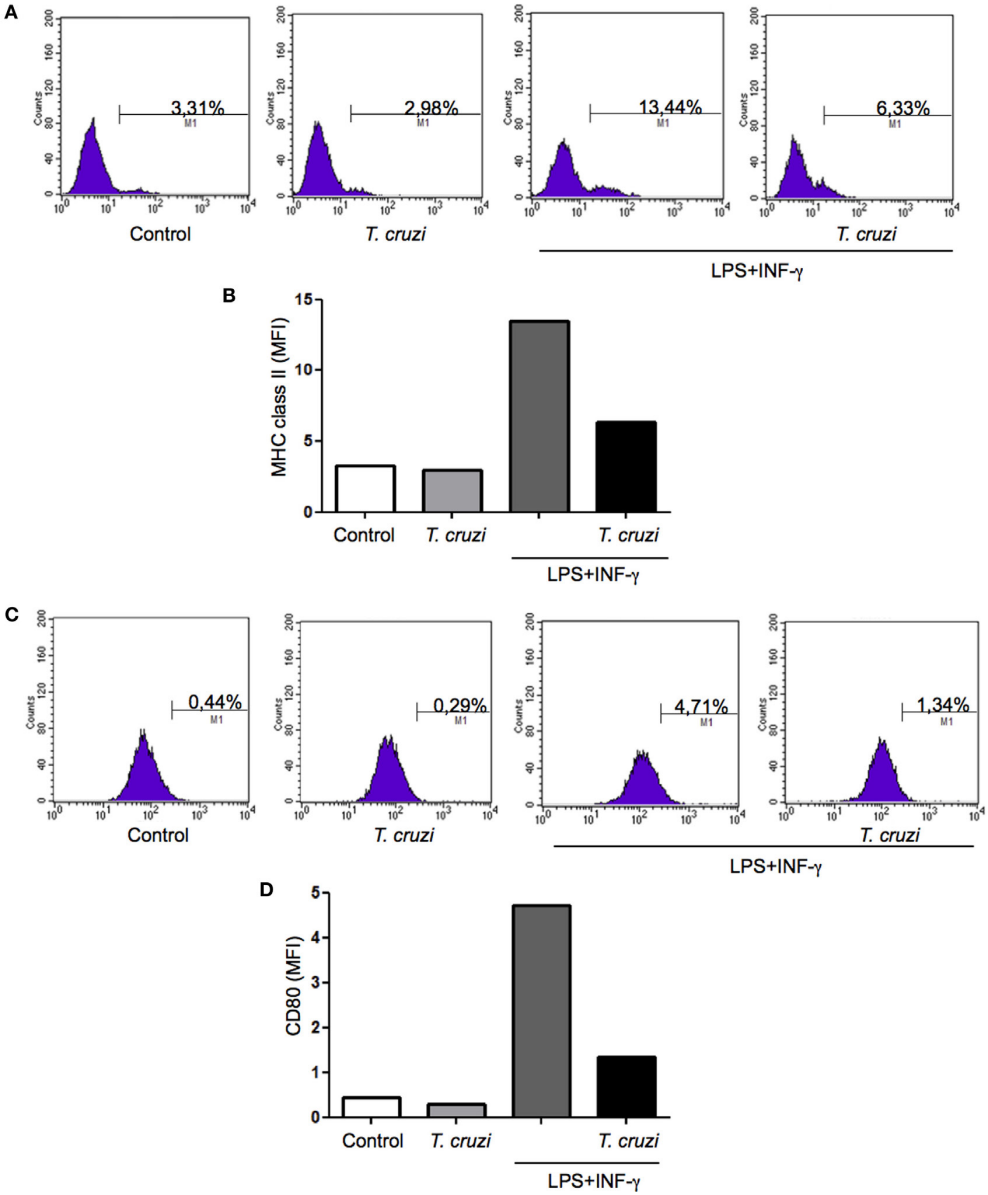
**Measurement of MHC class II and CD80 expression in infected DH82 by *Trypanosoma cruzi***. DH82 macrophages (5.0 × 10^5^/mL) were infected with trypomastigotes forms of *T. cruzi* Dm28c clone. After infection the cells were washed and some cultures were stimulated with LPS (400 ng/mL) and INF-γ (1.5 ng/mL). After 24 h, the cells were prepared for analysis by flow cytometry. The M1 marker shows the percentage of cells identified by MHC calss II **(A,B)** or CD80 **(C,D)**. The data are representative of three independent experiments.

The ability to express MHC class II and CD80 on surface confer to macrophage the status of antigen-presenting cell (APC). To be efficient APC during infection is very important to maintain unchanged the expression levels of these molecules (32). In order to study possible alterations resulting from *T. cruzi* infection that could lead to any modification in these molecules expression, DH82 canine macrophage cell line was infected or not and cultured in the presence or not of some activating factors, such as LPS and IFN-γ. After 24 h in culture, cells were harvested and labeled with monoclonal antibodies directed against MHC-II and CD80 and the expression was evaluated by flow cytometry analysis. The results showed that upon stimulation, the levels of surface MHC-II molecules increased but when DH82 macrophages were infected with *T. cruzi* the cells expressed lower levels of MHC II (Figures 6A,B). Upregulated surface expression of CD80 was also observed in DH82 stimulated and uninfected cells. On the other hand, low level of CD80 expression could be detected in *T. cruzi*-infected cells (Figures 6C,D). These data suggest that parasite infection inhibit the ability of DH82 canine macrophage cells to up regulate the expression of both molecules crucial to act as effective APCs.

Taken together, our results indicate that the DH82 canine macrophage cell line is susceptible to infection by *T. cruzi*, able to sustain the infection for long time when compare with RAW264.7, a murine macrophage cell line, support replication and differentiation to the parasite, culminating with the release of the infective forms that can be used as *in vitro* study to explore parasite-host interaction. Furthermore, the infection of activated cells induced production of cytokines known as deactivating factors for macrophage like TGF-β and IL-10, that when associated with decreased expression of MHC II and CD80 represent a powerful mechanism to modulate parasite growth in this cell line.

### Interaction between DH2 and Apoptotic Cells Increases Susceptibility to *T. cruzi* Infection

**Figure 7:**
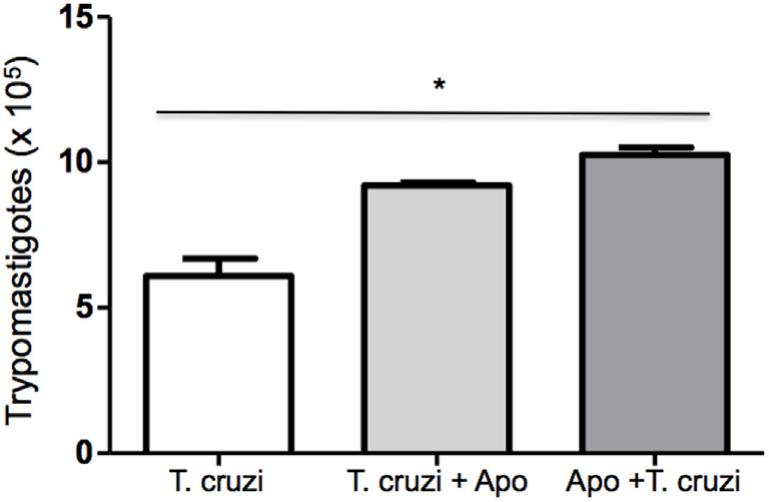
**Coculture of apoptotic cells with DH82 cells exacerbates infection by *Trypanosoma cruzi***. DH82 macrophages (2.5 × 10^5^/mL) were exposed to apoptotic T cells (five apoptotic cells for each DH82) 1 h before infection (Apo + *T. cruzi*) or infected and exposed to apoptotic T cells (*T. cruzi* + Apo). After 9 days, the trypomastigotes forms in the supernatants were counted. All cultures were performed in triplicate and bars show the mean + SD. Statistical analysis was performed by *t*-test from representative results of three similar experiments (*p ≤ 0.05).

Phagocytosis of apoptotic cells by *T. cruzi*-infected macrophages has the potential to down modulate macrophages leading to an anti-inflammatory response that results in increased parasite replication (22). In this context, we tested the modulatory effect of apoptotic cells on *T. cruzi*-infected DH82 canine macrophages. DH82 cell monolayers were cocultivated with apoptotic Jurkat T cells before or after infection with *T. cruzi*. As seen in Figure 4, the quantification of viable parasites released in the supernatants was made 9 days after infection. Cultures that received apoptotic cells before or after *T. cruzi* infection showed a significant increase in the number of released parasites (Figure 7), indicating similar modulation as previously described for mouse peritoneal macrophages.

## Discussion

Different cultured cells from mammal origin have been extensively used *in vitro* to study host-parasite interaction (33). Various studies have described the use of murine macrophage cell lines to analyze the infection of *T. cruzi* strains *in vitro* (34, 35) but infections of canine macrophages have not been evaluated. In the present work, we investigated whether the canine macrophage cell line DH82 is susceptible to *T. cruzi* infection *in vitro*. To determine the DH82 cells susceptibility to *T. cruzi* Dm 28c infection, macrophages monolayers were infected with metacyclic trypomastigotes differentiated *in vitro*. Our results showed that DH82 cells experienced infection rate of 30%. We found that trypomastigotes differentiated in amastigotes forms, replicated, and transform in trypomastigote before being released in cultures supernatants. To compare intracellular parasitism of *T. cruzi* in macrophages from a different host we also used mouse RAW264.7 tissue cultured macrophages. Microscopically, trypomastigotes free in the supernatants were detected earlier in DH82 than in RAW264.7 cells, as well as the number of parasites was higher in canine than in mouse macrophages. We also noted that DH82 macrophages were able to support infection for a prolonged period of time. This finding favors the use of DH82 cells as a model to study the *in vitro* interaction between *T. cruzi* and canine macrophages. The current study is the first to describe that DH82 cells are infected by *T. cruzi*, reproduce intracellular parasite replication, transformation into trypomastigotes and can be used as model to investigate host-pathogen interaction *in vitro* or can contribute in experiments to identify new drugs for the treatment of Chagas’ disease.

In the presence of the cytokines or bacterial products such as LPS or CPG DNA, macrophages exhibited an increase in NO and ROS production that represent a key defensive element in various infectious disease (36). In *in vitro* models of macrophages infection by *T. cruzi*, the resistance to infection is closely associated with activation and consequent increase in the microbicidal activity of macrophages (37, 38). Our results showed that resistance of the DH82 cells to *T. cruzi* infection was increased after stimulation, indicating that pretreatment with classical activators can modulate these macrophages. An opposite effect was seen when *T. cruzi*-infected DH82 cells were cocultured with apoptotic lymphocytes. After interact with apoptotic cells canine macrophages became more susceptible to infection, independently if apoptotic lymphocytes were added to the culture before or after infection. Interaction with apoptotic cells lead macrophages to secrete TGF-β, which in turn suppresses their proinflammatory cytokine response. In addition, TGF-β renders both phagocytic and non-phagocytic cells permissive to *T. cruzi* infection (39) and antagonizes INF-γ-induced NO production and macrophage trypanocidal activity (22). Based on these, ours results suggest that canine macrophage DH82 can be driven to the resistance or susceptibility depending on stimulus received that might increase their microbicidal activity or become permissive to cell invasion.

Since our results showed that DH82 cells become more resistant to infection after being stimulated with LPS and IFN-γ, we investigated which molecules could be involved. We attempted to quantify NO production; however, the cells were not able to increase NO even after stimulation with LPS and INF-γ (Figure S1 in Supplementary Material). Similar results were described in a previous study (40) that shown unchanged levels in NO production by DH82 cells. On the other hand, ROS production by canine macrophages was evident after stimulation. In our model, *T. cruzi*-infected cells were capable of producing ROS at similar levels detected in stimulated cells only and the production was markedly enhanced in the presence of LPS + IFN-γ. Despite the production of high levels of ROS by *T. cruzi*-infected DH82 macrophages, intracellular parasite replication was not effectively affected. It is noteworthy to mention that a recent report showed that to withstand such variable sources of oxidative stress, *T. cruzi* has developed complex defense mechanisms. This includes ROS detoxification pathways that are distinct from the ones in the mammalian host, DNA repair pathways and specialized polymerases, which not only protect its genome from the resulting oxidative damage but also contribute to the generation of genetic diversity within the parasite population (41).

When stimulated DH82 macrophages were infected, we detected a strong increase in TNF-α production accompanied by TGF-β and IL-10. This profile suggests that DH82 canine macrophages infected by *T. cruzi* are modulated to produce anti-inflammatory cytokines and this phenomenon can be increased in stimulated cells. In fact, high levels of TGF-β were detected in stimulated and infected cells, as well as in uninfected cells. TGF-β is an important anti-inflammatory mediator and participates in the metabolism of l-arginine, which favors the production of arginase and is involved in the activity of ornithine decarboxylase. The intracellular stage of *T. cruzi* requires this host cell enzyme and the synthesis of polyamines to multiply inside the cell (42). We suggest that this mechanism is involved in the successful infection of DH82 canine macrophage by *T. cruzi*.

Protozoan parasites such as *T. cruzi* can modulate the function of dendritic cells by interfering with recognition, migration, and antigen presentation (43). It is known that *T. cruzi* inhibits the expression of MHC II, CD40, CD80, and CD86, hampers the production and secretion of IL-12, TNF-α, and IL-6, increases the production of the cytokine IL-10, and inhibits antigen presentation in murine and human dendritic cells *in vitro* (44). Corroborating the *in vitro* findings, *in vivo* experiments have also demonstrated that *T. cruzi* is able to impair many aspects of dendritic cells biology. Additionally during acute infection, splenic dendritic cells migration and expression of the CD86 are inhibited in infected mice with *T. cruzi* (45). Interestingly, the same correlation was observed in our study with DH82 canine macrophages. These cells showed a reduction in MHC II and CD80 expression following infection with *T. cruzi*. The MHC II and CD80 expression was increased in LPS and IFN-γ stimulated cells, while infection by *T. cruzi* affected the ability of previously activated cells to increase MHC II and CD80 expression in DH82 cells. Taken together, our results reinforce the relevance of *T. cruzi* infection in modulating canine macrophages and the role in the infection establishment and disease progression. In addition, our results suggest that canine macrophages DH82 are more susceptible to infection by *T. cruzi* when compared with RAW264.7 murine macrophages.

## Author Contributions

DD-R, CGF-de-L, and PHBM conceived and designed the experiments. PHBM, RFDBR, JBBM, IFLRF, and JL performed the experiments. DD-R, CGF-de-L, MPN, and AM analyzed the data. DD-R and CGF-de-L wrote the article.

## Conflict of Interest Statement

The authors declare that the research was conducted in the absence of any commercial or financial relationships that could be construed as a potential conflict of interest. The handling editor declared a shared affiliation, though no other collaboration, with several of the authors LF, JL, CL and states that the process nevertheless met the standards of a fair and objective review.

## Supplementary Material

Figure S1 **DH82 canine macrophage do not upregulate nitric oxide**. RAW264.7 and DH82 macrophages were cultured (2.5 × 10^5^/mL) in the absence or presence of LPS (200 or 400 ng/mL) and INF-γ (1.5 ng/mL). After 12 and 24 h of incubation, NO production was evaluated by Griess colorimetric method. All cultures were performed in triplicate and bars show the mean + SD. Statistical analysis was performed by *t*-test from representative results of three similar experiments (****p* ≤ 0.0001).
