# Supplementary figures and images for: Canine Macrophage DH82 Cell Line As a Model to Study Susceptibility to Trypanosoma cruzi Infection
Source: Front Immunol. 2017 May 31;8:604. doi: 10.3389/fimmu.2017.00604 (PMC5449653; doi:10.3389/fimmu.2017.00604)

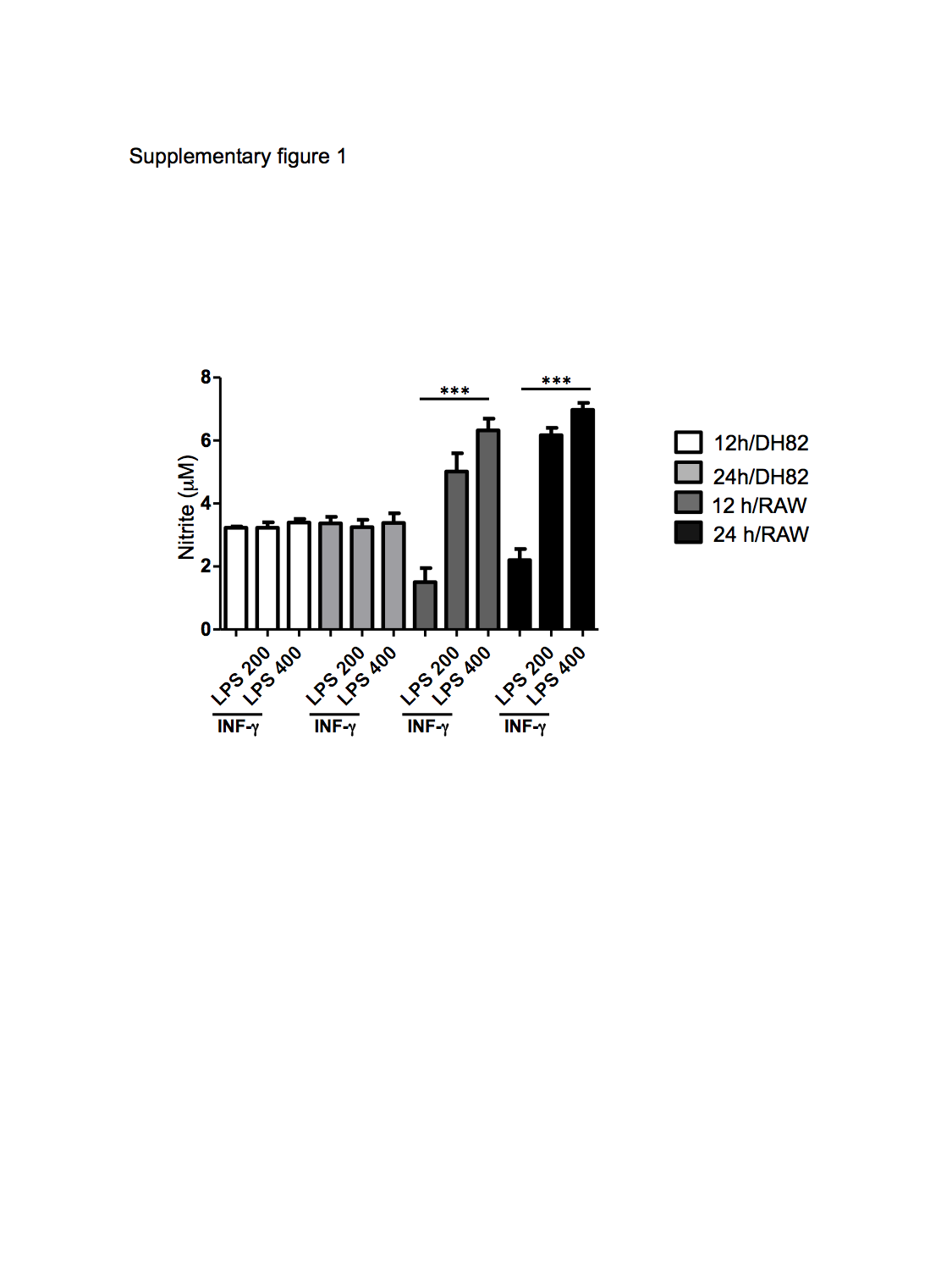

Supplement: Figure S1 — DH82 canine macrophage do not upregulate nitric oxide. RAW264.7 and DH82 macrophages were cultured (2.5 × 10^5^/mL) in the absence or presence of LPS (200 or 400 ng/mL) and INF-γ (1.5 ng/mL). After 12 and 24 h of incubation, NO production was evaluated by Griess colorimetric method. All cultures were performed in triplicate and bars show the mean + SD. Statistical analysis was performed by t-test from representative results of three similar experiments (***p ≤ 0.0001). [file Image_1.tif]
